# Supplementary material for: The epichaperome is a mediator of toxic hippocampal stress and leads to protein connectivity-based dysfunction
Source: Nat Commun. 2020 Jan 16;11:319. doi: 10.1038/s41467-019-14082-5 (PMC6965647; doi:10.1038/s41467-019-14082-5)
Supplement: Supplementary file 1 — Supplementary Information [file 41467_2019_14082_MOESM1_ESM.pdf]

The epichaperome is a mediator of toxic hippocampal stress and leads to protein connectivity-based dysfunction

Inda MC, Joshi S, Wang T, Bolaender A, et al.

Contains Supplementary Figures 1 through 8

A

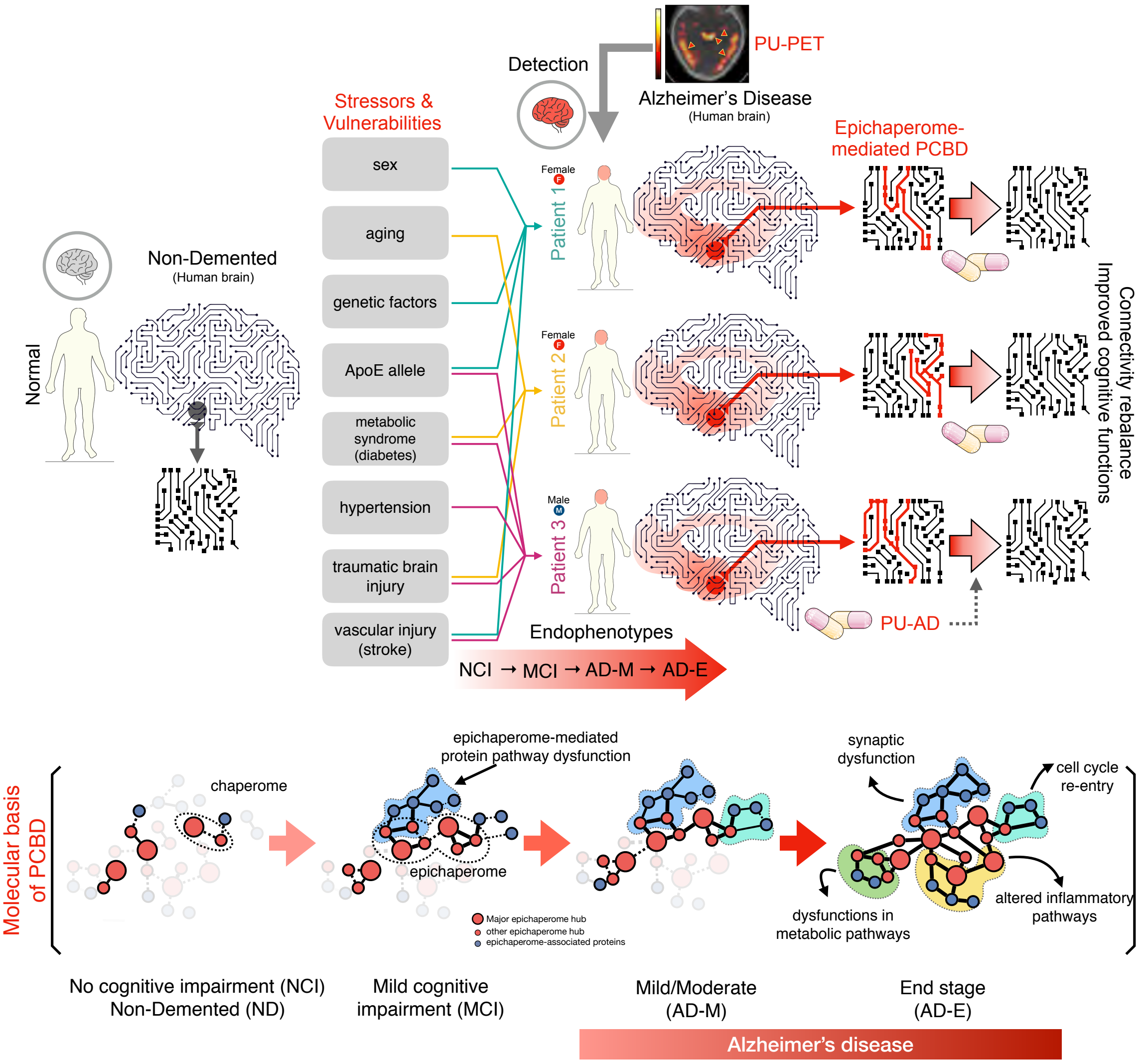

B

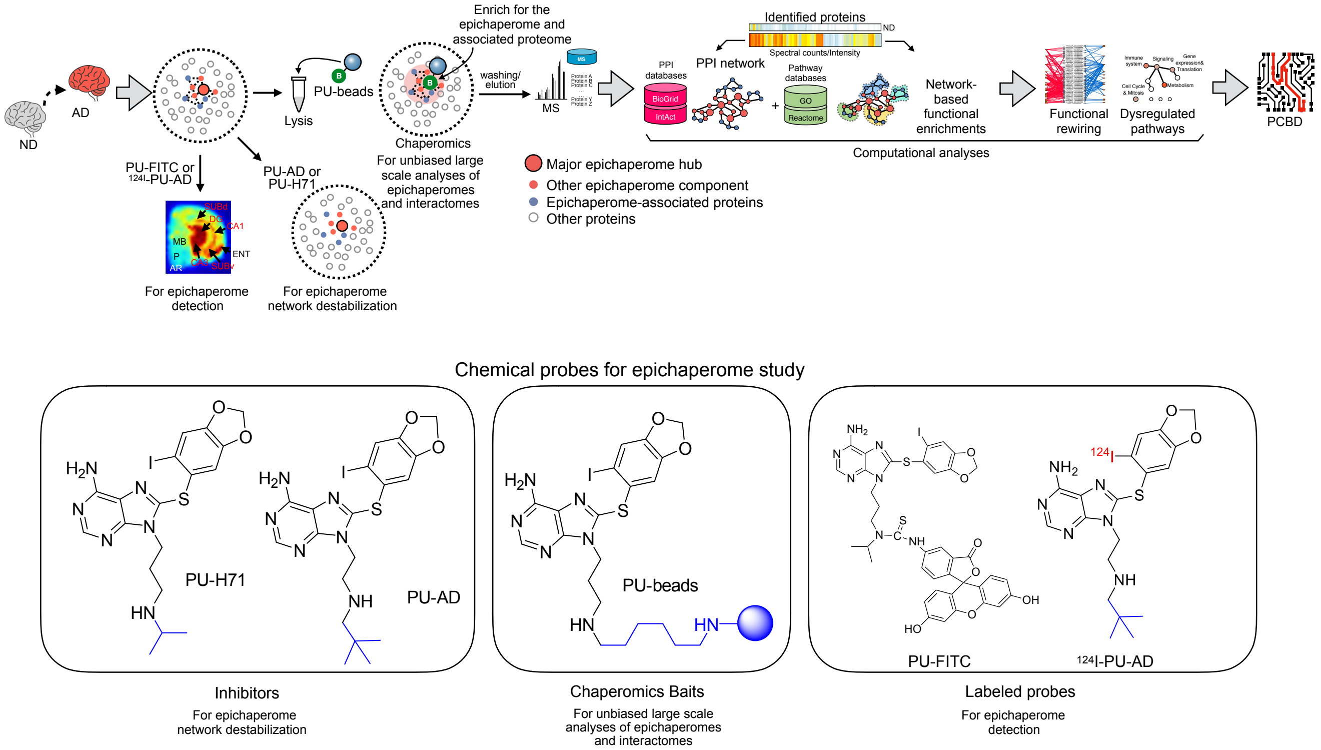

Supplementary Fig. 1 Working hypothesis of the present study and its translational and transformative potential.

(***Continued next page***)

Supplementary Fig. 1 (*continued*)

A We propose a mechanism whereby normal functioning chaperomes within vulnerable brain regions reconfigure to pathological epichaperomes that appear to underlie neurodegenerative disorders with significant stress-related pathobiology, including AD. We propose the switch of the chaperome into epichaperomes supports proteome-wide connectivity dysfunction, and that such dysfunction progresses into global disturbances in dynamic intra- and inter-neuronal networks, and ultimately reverberates at a target organ level. We posit that functionally AD is a protein connectivity based dysfunction (PCBD), a previously unappreciated aspect of AD biology. Specifically, a functional outcome of protein connectivity disruption occurs in vulnerable neocortical regions directly leading to cognitive decline, especially in memory and executive function domains. The present study proposes a linear paradigm shift whereby structural changes in the epichaperome directly lead to deleterious functional outcomes in terms of PCBD, and we provide evidence for how protein connectivity dysfunctions alter protein-to-neuronal circuit-to-organ level changes in the context of AD. Thus, AD can be considered a disorder whereby dynamic protein networks are rewired, which leads to pathological changes in brain circuitry (both loss of normal function and gain of aberrant function).

From a translational perspective, epichaperomes, and in turn the associated protein connectivity disruption, are targetable in vivo through delivery of a therapeutic, PU-AD, in clinical evaluation first in healthy volunteers, then in AD patients (NCT03935568). We provide proof-of-principle that PU-AD reverses synaptic network imbalances, and improves learning and memory. From a transformative perspective, imaging with PU-AD PET-CT reveals epichaperomes in living patients diagnosed with AD and not in age-matched cognitively normal controls. This exciting finding may prove to be a therapeutic strategy, alone or in combination with other proposed AD-pathology reducing treatments, that corrects one of the basic hallmarks of AD, namely synaptic dysfunction within vulnerable memory and executive function circuits. This provides a proof-of-concept for a new avenue of rational therapeutics aimed at correcting PCBD through de-connecting aberrant epichaperome structures and the proteome-wide pathways they rewire, especially those that drive synaptic dysfunction in vulnerable areas critical for normative cognitive processes including memory and executive function.

B This work applies novel, unbiased large-scale chemical biology and bioinformatics analyses pipelines and chemical probes (ex. PU-AD, PU-H71, ^{124}I-PU-H71, PU-FITC and PU-beads) to uncover a new mechanism of disease – protein connectivity dysfunction – and then validates these provocative findings in cellular and animal models as well as in humans (both in live patients and postmortem biospecimens). In this chemical biology approach to AD, PU-H71 and PU-AD are used to inhibit the epichaperome, and in turn probe the dependence of proteome alterations on the epichaperome. PU-beads are used to isolate the epichaperome and its interactome, which are then analysed unbiasedly through mass spectrometry and a bioinformatics pipeline to inform on the function of epichaperome-mediated proteome alterations (i.e. the basis of chemical chaperomics). PU-FITC is used to identify the epichaperome through fluorescence polarization, fluorescence microscopy or flow cytometry, whereas radiolabeled PU-AD is used to detect the epichaperome by autoradiography (^{131}I-PU-AD) or by positron emission tomography (^{124}I-PU-AD for PU-AD PET).

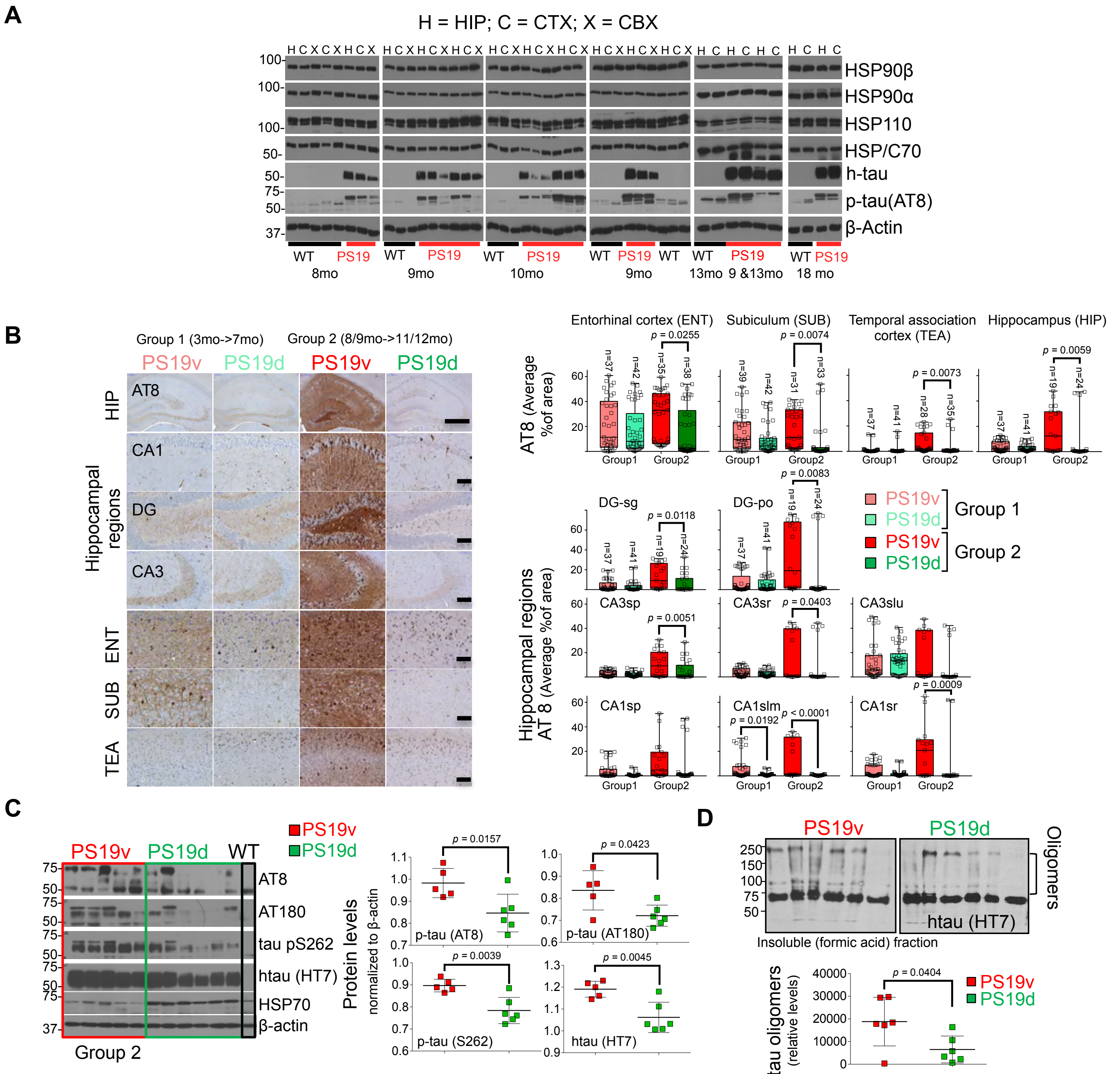

Supplementary Fig. 2 Tau pathology and its relationship to the epichaperome. **A** Age-dependent expression of tau, hyperphosphorylated tau and individual chaperome members within indicated brain regions of WT (n = 8) and age-matched PS19 (n = 6) mice. H = hippocampus; C = frontal cortex; X = cerebellum. **B** Representative AT8 immunostaining of indicated brain areas and graphed data for PS19 mice treated with vehicle or PU-AD, as indicated in Fig. 8A. Epichaperome inhibition in PS19 mice blocked the formation of AT8 human tau species (see PU-AD treatment of mice from 3 mo to 7 mo of age) but also reduced the already formed AT8 species (see PU-AD treatment of mice from 8-9 mo to 11-12 mo of age). Graph, mean ± SEM one-way ANOVA, Tukey's post-hoc. Slides (n as indicated for each group) were counterstained with hematoxylin. Scale bars, 200µm for HIP, 50µm for all other. **C**,**D** Western blot showing the expression of soluble tau species **C** and insoluble (FA extracted, panel **D**) in individual AD mice treated with vehicle or PU-AD, as indicated in Fig. 8A (Group 2). β-actin, loading control. WT, non-Tg control for mouse tau and antibody cross-reactivity. Graph, mean ± SEM, unpaired two-tailed t-test, with Welch's correction, n = 5 vehicle, n = 6 PU-AD for panel **C** and n = 6 vehicle, n = 6 PU-AD for panel **D**. Source data are provided as a Source Data file.

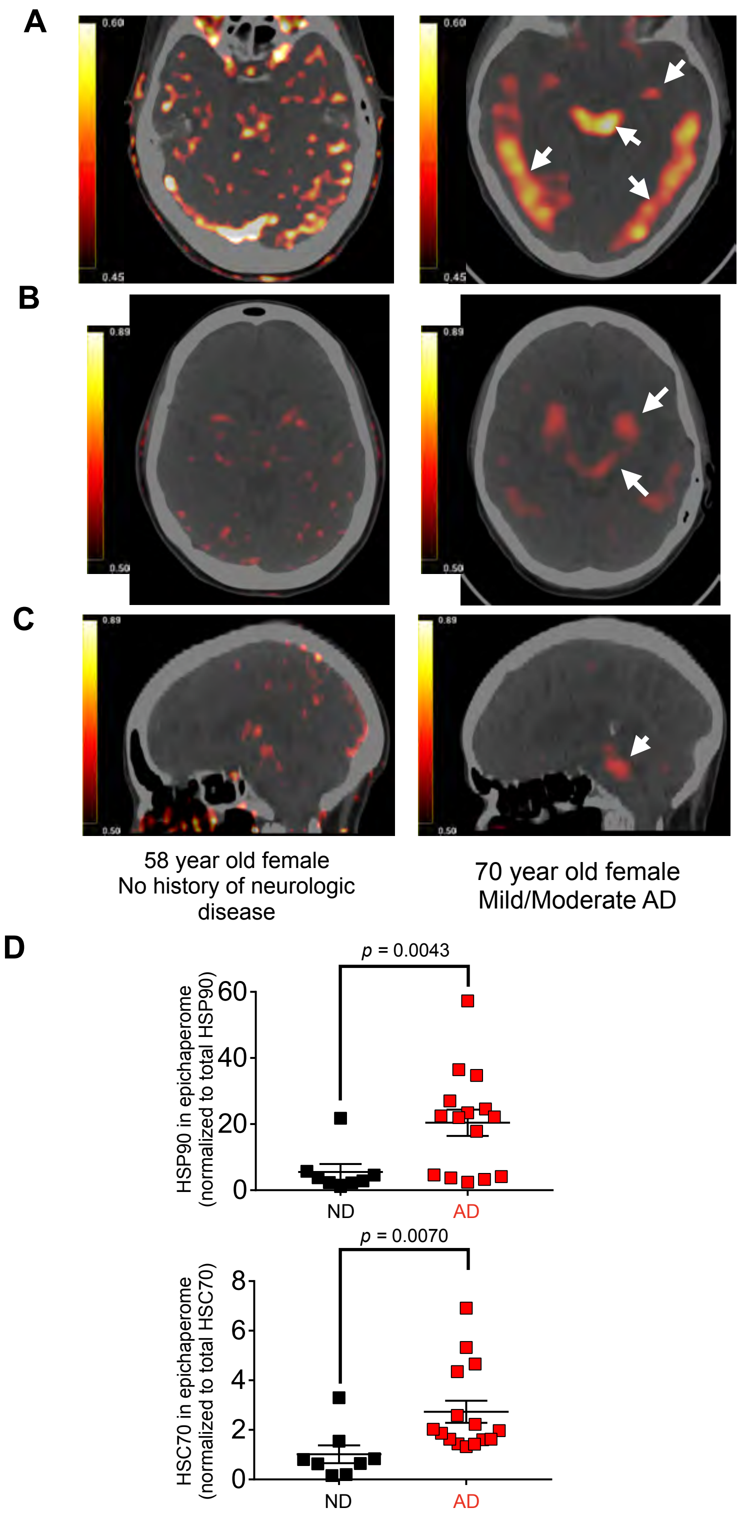

Supplementary Fig. 3 The epichaperome is specific to the AD brain and is independent of the expression levels of individual units. **A**-**C** ^{124}I-PUAD PET-CT image sets from two different patients show the spatial epichaperome expression within the live human brain. **A**,**B** show corresponding axial planes through the brain, and **C** sagittal planes through the brainstem. Images were performed 3 h after ^{124}I-PU-AD tracer injection. The AD patient's bilateral parahippocampal region (e.g., left uncus, arrow), brainstem (arrow) and bilateral temporal lobes (arrows) demonstrate high radiotracer retention relative to the control patient, indicating epichaperome expression. Thalami (arrows) and basal ganglia (arrows) also show mild tracer uptake. The control patient, with no history of neurologic disease or cognitive decline, demonstrates no epichaperome signal above background (i.e. noise artifact). Numbers in the scale bars indicate upper and lower SUV thresholds that define pixel intensity on PET images. PET, positron emission tomography; CT, computerized tomography. **D** Same as in Fig. 2B with epichaperome levels normalized to total chaperone expression levels. Graph, mean ± SEM, unpaired two-tailed *t*-test, with Welch's correction, n = 15 for AD and n = 8 for ND. Source data are provided as a Source Data file.

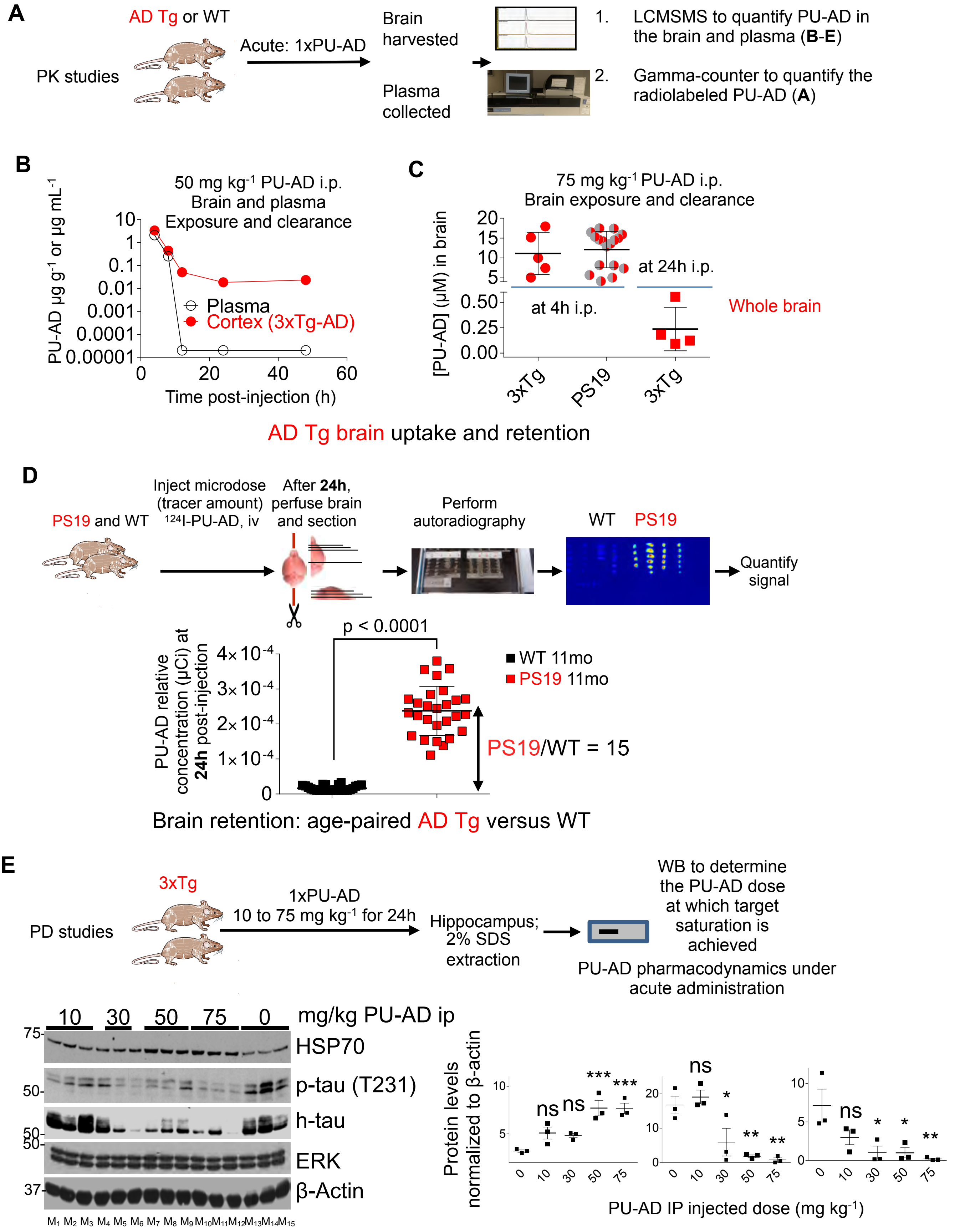

Supplementary Fig. 4 Characterization of PU-AD: pharmacokinetics and pharmacodynamics in normal and AD brains. **A**-**D** AD and WT mouse brain exposure after single-dose PU-AD. In the normal brain, the residence time of PU-AD was less than 4 h, and similar to that seen in plasma, while in the AD brain (i.e. 3xTg and PS19), PU-AD was retained for over 24 h. At 24 h after injection, the brain concentration of PU-AD in the 3xTg mice was 250 nM (i.e. 50-fold greater than its affinity for the epichaperome measured in these mice). At this time, PU-AD was largely undetected in plasma (**B**) or in the normal brain (**D**).

Continued next page

Supplementary Fig. 4 (*continued*)
D A radiolabeled PU-AD (^{131}I-PU-AD or ^{124}I-PU-AD, tracer amount) was injected i.v. and PU-AD levels were determined by autoradiography at 24 h post injection in brain sections obtained from PS19 and WT mice (11mo F), to demonstrate specific AD brain retention of PU-AD. The retention of PU-AD in the AD brain is due to kinetic selectivity for epichaperomes with a long binding half-life (slow k_{off}). i.p., intraperitoneally, i.v. intravenously. **B** Each data point represents the mean of 3 individual mice (8-10mo M 3xTg, *n* = 15). **C** Error bars show mean ± SD. **D** Error bars show mean ± SEM, unpaired two-tailed *t*-test, PS19 (*n* = 27) and WT (*n* = 26). $P < 0.0001$. See also Fig. 1 for the anatomical localization of PU-AD in the PS19 brain and Supplementary Fig. 3, in a representative human AD brain. **E** Inhibition of hyperphosphorylated tau was observed at doses of PU-AD ranging from 30 to 75 mg kg^{-1} with saturation reached at 50-75 mg kg^{-1} under i.p. administration, which corresponds to the dose we also observed maximal epichaperome suppression by PU-AD (see Fig. 7B). 12-months old (mo) 3xTg (male) M mice were administered the indicated doses of PU-AD and mice were sacrificed 24 h post single injection. Each data point represents an individual mouse (M_1 through M_n). Error bars show mean ± SEM, one-way ANOVA, Dunnett's post-hoc, *n* = 3 per dose. ****$P < 0.0001$; ***$P < 0.001$; **$P < 0.01$; *$P < 0.05$. Source data are provided as a Source Data file.

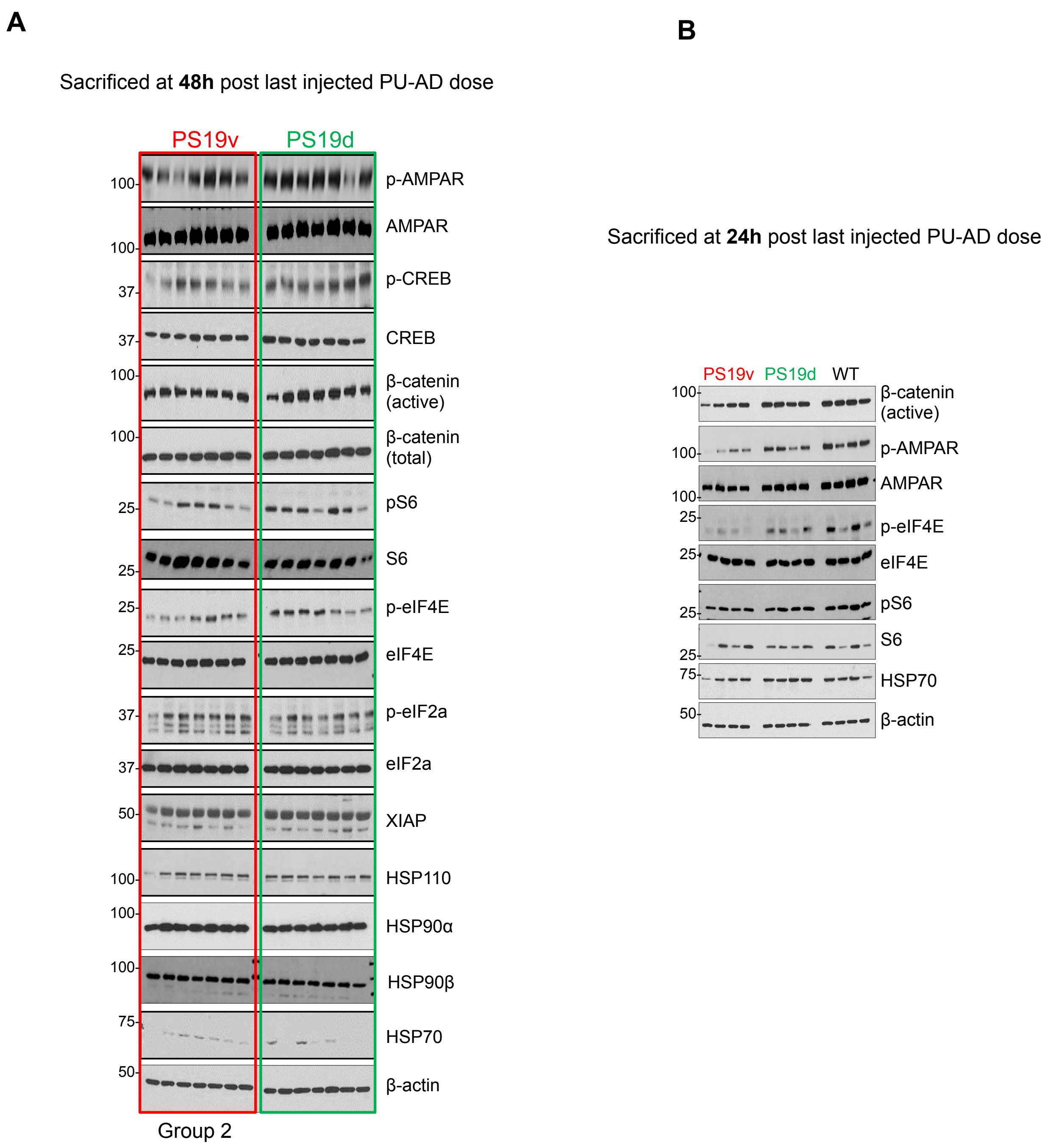

Supplementary Fig. 5 Epichaperome inhibition restores the function of synaptic protein pathways in PS19 mice to WT levels. **A** Western blot of PS19 mice (hippocampus) treated with vehicle or PU-AD as indicated in Fig. 8A. Mice were sacrificed 48h post-last injected dose. See also Fig. 7C. **B** A rebalance in the function of synaptic protein pathways to WT levels is observed in PS19 mice treated with PU-AD. Same as in **A** for mice sacrificed at 24h post-last injected dose of the 6-week treatment paradigm. See also Fig. 7D. Source data are provided as a Source Data file.

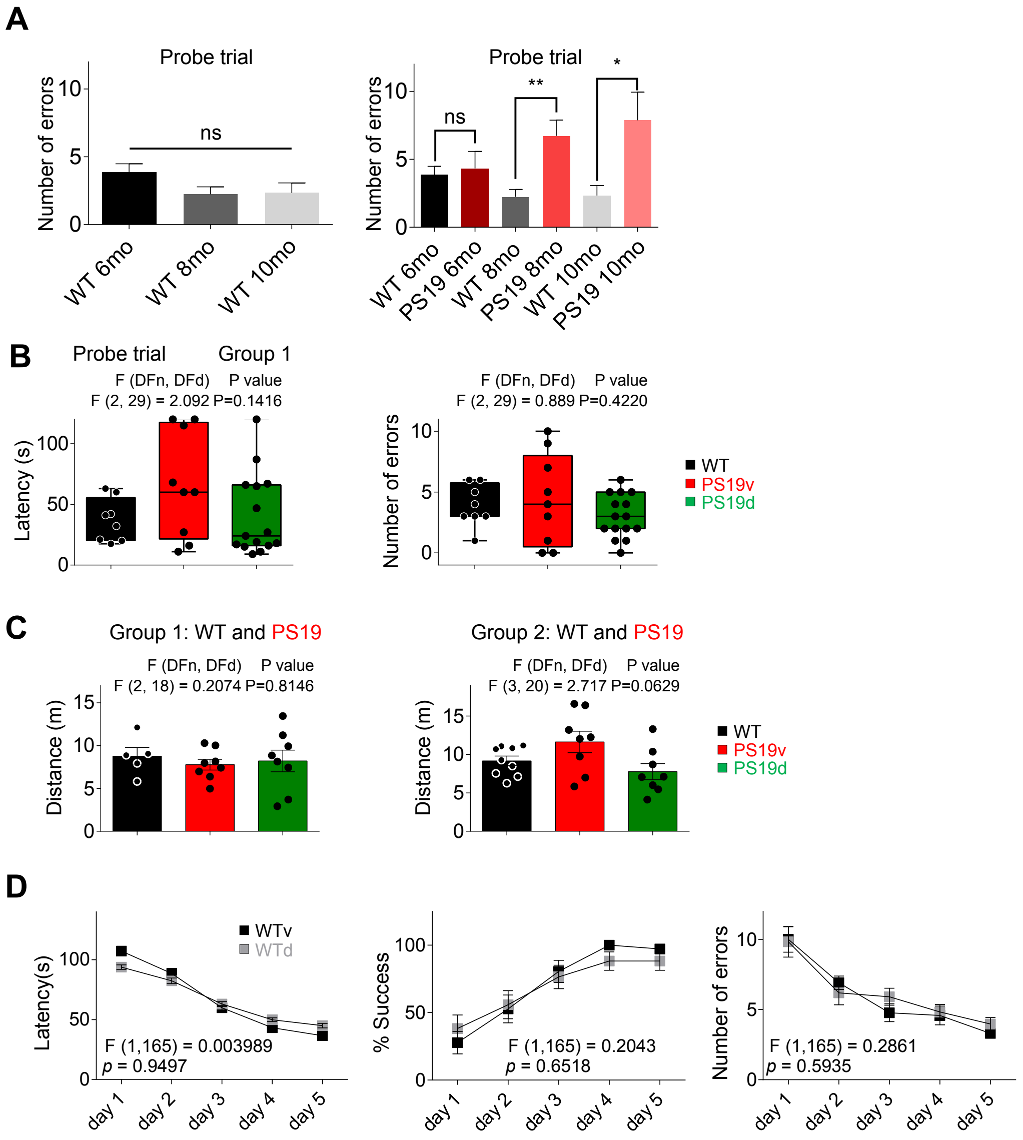

Supplementary Fig. 6 Behavioral studies in PS19 mice. **A** Cognitive defects associated with hippocampal function in PS19 mice at ages 6, 8 and 10-11 mo, specifically in spatial learning and memory, were observed using the Barnes maze. This test requires mice to learn and remember how to escape the maze using visual cues. All WT mice learned to locate the escape hole through training, as indicated by a progressive reduction in the number of errors made before identifying the escape hole (measured as 'Number of errors'). When compared to WT, PS19 mice demonstrated an increase in the number of errors, a deficit that became more robust with age (probe memory retention trial: P = 0.7557 and P = 0.0017, at 6 mo and 8 mo, respectively). Error bars show mean ± SEM, unpaired t-test, wild-type (WT) mice, n = 8, 17 and 9 for 6, 8 and 10 mo, respectively, and PS19 n = 9, 18 and 8 for 6, 8 and 10 mo, respectively. **B** Spatial learning and memory evaluated in the Barnes maze in vehicle or PU-AD treated PS19 mice (PS19v and PS19d, respectively), and WT mice (Group 1, see also Fig. 8A). Probe memory-retention trial was performed 24h after the last acquisition session. Error bars show mean ± SEM, one-way ANOVA; n = 8 WTv, n = 9 PS19v, n = 15 PS19d. **C** Open field locomotor activity. PS19 and WT mice treated with PU-AD or vehicle as described in Fig. 8A were monitored for 4 min and distance traveled was recorded. Error bars show mean ± SEM, one-way ANOVA with Dunnett's post-hoc, Group 1: WTv, n = 5; PS19v, n = 8; PS19d, n = 8; Group 2: WTv, n = 9; WTd, n = 9; PS19v, n = 8; PS19d, n = 8. **D** Spatial learning and memory evaluated in the Barnes maze following PU-AD or vehicle treatment of WT mice. Error bars show mean ± SEM, two-way ANOVA, n = 18 WTv, n = 17 WTd. Pooled data from 2 experiments. Source data are provided as a Source Data file.

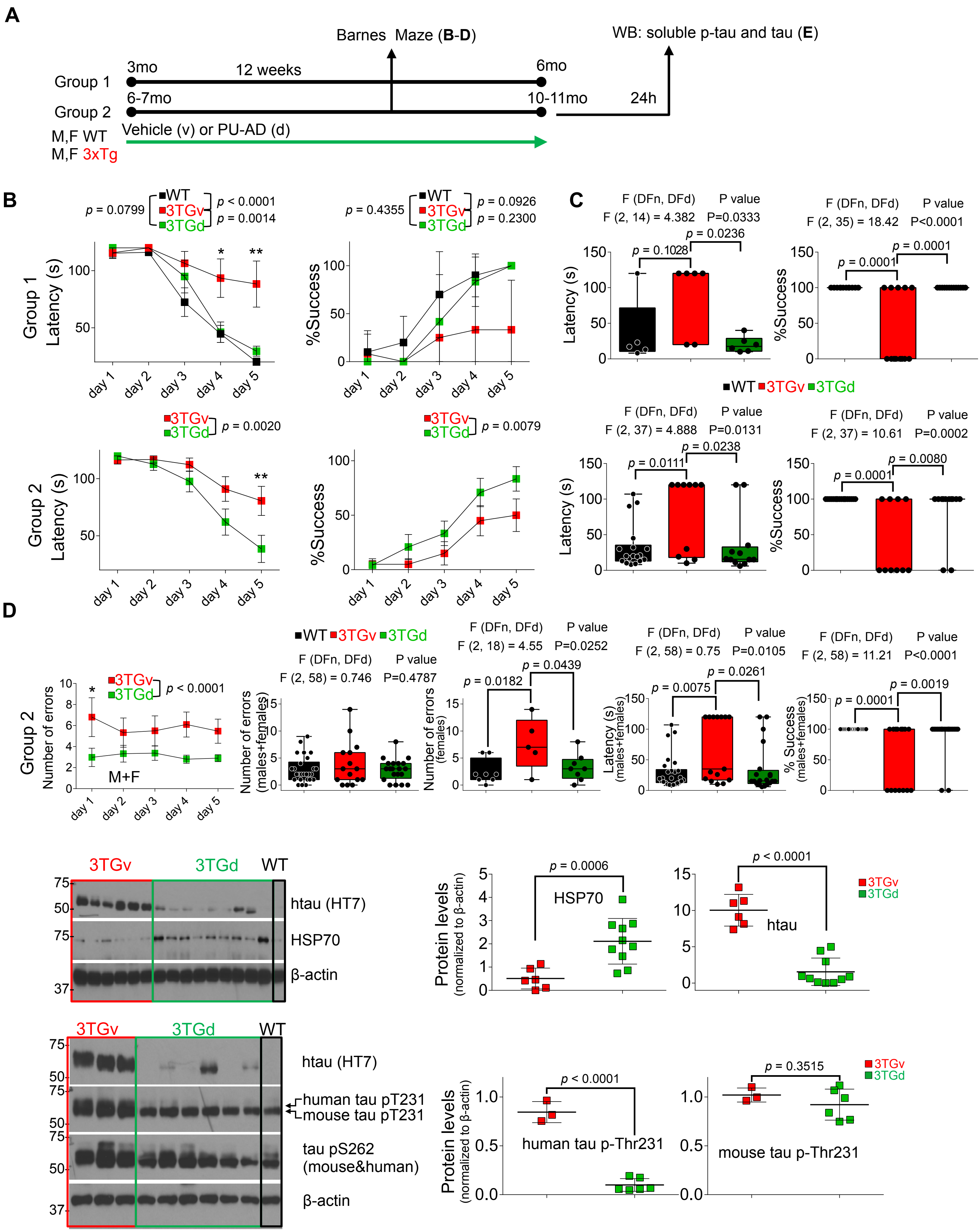

Supplementary Fig. 7 Epichaperome inhibition restores spatial learning and memory in 3xTg mice.

(*Continued next page*)

Supplementary Fig. 7 (*continued*)
A Schematic of the treatment and testing paradigm. **B**,**C** 3xTg mice demonstrate impaired spatial memory recorded in the Barnes maze. PU-AD treatment restores memory in 3xTg mice to WT levels. **B** Acquisition phase: Error bars show mean ± SEM, two-way ANOVA, Bonferroni post-hoc. **C** Probe memory retention trial: Error bars show mean ± SEM, one-way ANOVA with Dunnett's post-hoc. Group 1, males. n = 6 vehicle treated 3xTg mice (3TGv), n = 6 PU-AD treated 3xTg (3TGd) and n = 5 vehicle treated (WTv). Group 2, males. n = 18 WTv, n = 10 3TGv and n = 12 3TGd. **D** Number of errors determined in the Barnes maze for 11mo 3xTg mice (males and females). Acquisition phase: Error bars show mean ± SEM, two-way ANOVA, $P < 0.0001$, F (1, 165) = 18.55; n = 10M+5F 3TGv and n = 12M+8F 3TGd. Probe memory retention trial: Error bars show mean ± SEM, one-way ANOVA with Dunnett's post-hoc, n = 18M+8F WTv, n = 10M+5F 3TGv and n = 12M+8F 3TGd. Pooled data from 3 experiments. **E** Western blot analysis showing the expression of human soluble tau species in individual mice treated with Vehicle or PU-AD as indicated in **A**. Error bars show mean ± SD, unpaired t-test with Welch's correction, n = 5 3TGv, n = 10 3TGd. In accord with a function of the epichaperome in mediating pathologic proteostasis changes, but normal proteostasis, we found normal mouse tau phosphorylated at Thr231 and Ser262 to be largely unaffected by PU-AD. WT mouse, non-Tg used as a control for mouse tau and antibody cross-reactivity; β-actin, loading control; Graph data, mean ± SD, unpaired t-test, n = 3 3TGv, n = 6 3TGd. Source data are provided as a Source Data file.

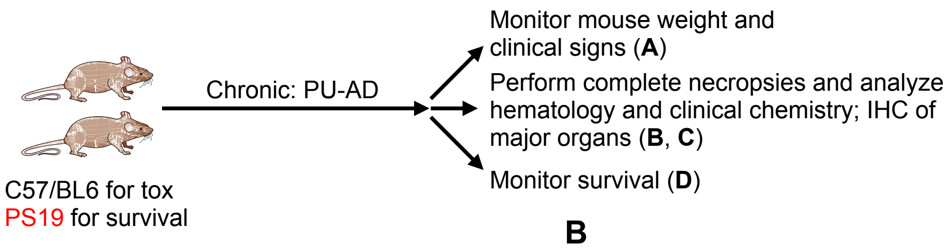

A

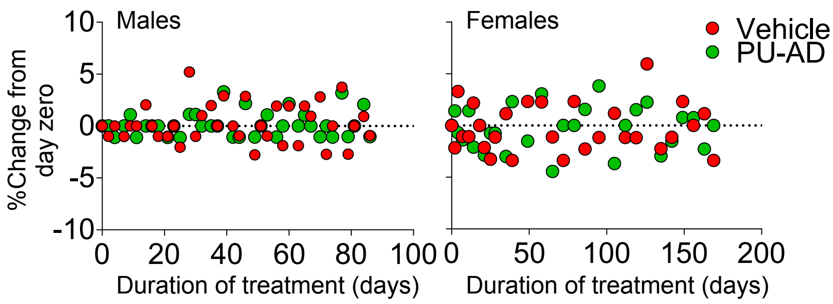

B

Parameter	Unit	Ref range	Vehicle	PU-AD 75 mg/kg
WBC	K µL^{-1}	1.8-10.7	7.31 +/- 3.6	4.9 +/- 2.0
NEUTROPHILS	K µL^{-1}	0.1-2.4	3.2 +/- 3.0	1.4 +/- 0.7
LYMPHOCYTES	K µL^{-1}	0.9-9.3	3.3 +/- 1.0	3.2 +/- 1.4
MONOCYTES	K µL^{-1}	0.0-0.4	0.6 +/- 0.34	0.2 +/- 0.07
EOSINOPHILS	K µL^{-1}	0.0-0.2	0.1 +/- 0.1	0.1 +/-0.05
BASOPHILS	K µL^{-1}	0.0-0.2	0 +/- 0.01	0 +/- 0.01
RBC	M µL^{-1}	6.36-9.42	9.6 +/- 0.62	9.4 +/- 0.54
HB	g dL^{-1}	11.0-15.1	13.9 +/- 0.7	13.5 +/-1.0
HCT	%	35.1-48.4	48.68 +/- 2.21	47.94 +/- 3.92
MCV	fL	45.4-60.3	50.6 +/- 1.88	50.9 +/- 3.11
MCH	Pg	14.1-19.3	14.48 +/- 0.45	14.34 +/- 0.75
MCHC	g dL^{-1}	30.2-34.2	28.6 +/- 0.41	28.2 +/- 0.55
RDW	%	12.4-27.0	23.2 +/- 1.58	24.2 +/- 1.30
PLT	K µL^{-1}	592-2972	1127.3 +/-192.92	1201.4 +/- 270.33
MPV	fL	5.0-20.0	6.3 +/-0.43	6.2 +/- 0.56
ALP	IU L^{-1}	23-181	86.8 +/- 52.09	80.8 +/- 21.76
ALT (SGPT)	IU L^{-1}	16-58	37.7 +/- 7.47	53.8 +/- 30.92
AST (SGOT)	IU L^{-1}	36-102	83.7 +/- 37.68	96.3 +/- 27.16
Albumin	g dL^{-1}	2.5-3.9	2.9 +/- 0.19	3.2 +/- 0.17
Total Protein	g dL^{-1}	4.1-6.4	5.7 +/- 0.34	5.8 +/- 0.53
Globulin	g dL^{-1}	1.3-2.8	2.8 +/- 0.30	2.6 +/- 0.40
Total Bilirubin	mg dL^{-1}	0.0-0.3	0.15 +/- 0.05	0.17 +/- 0.05
BUN	mg dL^{-1}	14-32	25.1 +/- 8.13	26 +/- 3.70
Creatinine	mg dL^{-1}	0.1-0.6	0.19 +/- 0.08	0.16 +/- 0.06
Cholesterol	mg dL^{-1}	74-190	116.3 +/- 35.06	123.6 +/- 28.85
Glucose	mg dL^{-1}	76-222	168 +/- 23.73	171.1 +/- 13.04
Calcium	mg dL^{-1}	7.6-10.7	9.8 +/- 0.23	10 +/- 0.47
Phosphorus	mg dL^{-1}	4.6-9.3	8.0 +/- 1.38	8.2 +/- 1.18
Chloride	mEq L^{-1}	103-115	109.9+/- 1.57	109 +/- 2.0
Potassium	mEq L^{-1}	3.4-5.5	7.7 +/- 1.27	7.7 +/- 0.46
Sodium	mEq L^{-1}	146-155	152.3 +/- 1.25	151.1 +/- 2.12
A/G ratio		1.0-2-2	1.1 +/- 0.15	1.2 +/- 0.16
Na/K Ratio		26.7-42.1	20.1 +/- 4.02	20 +/- 1.29

C

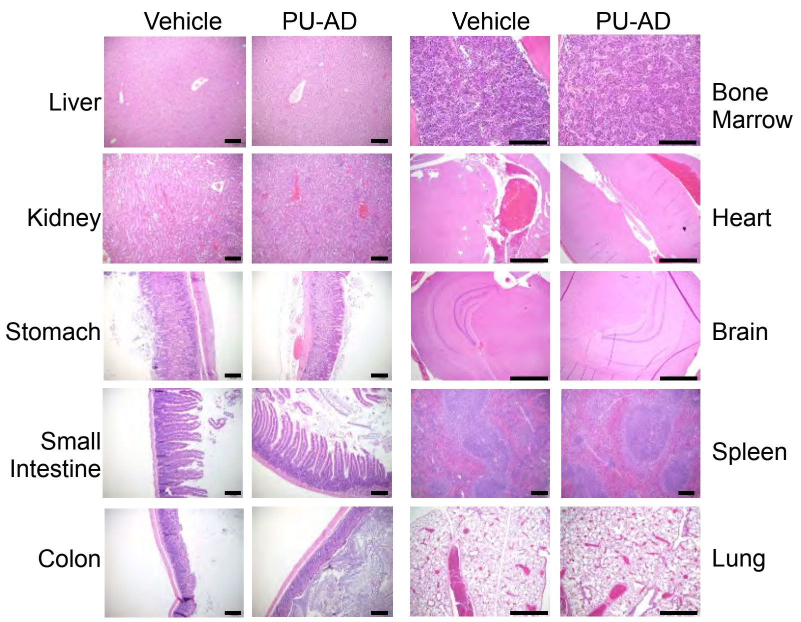

D

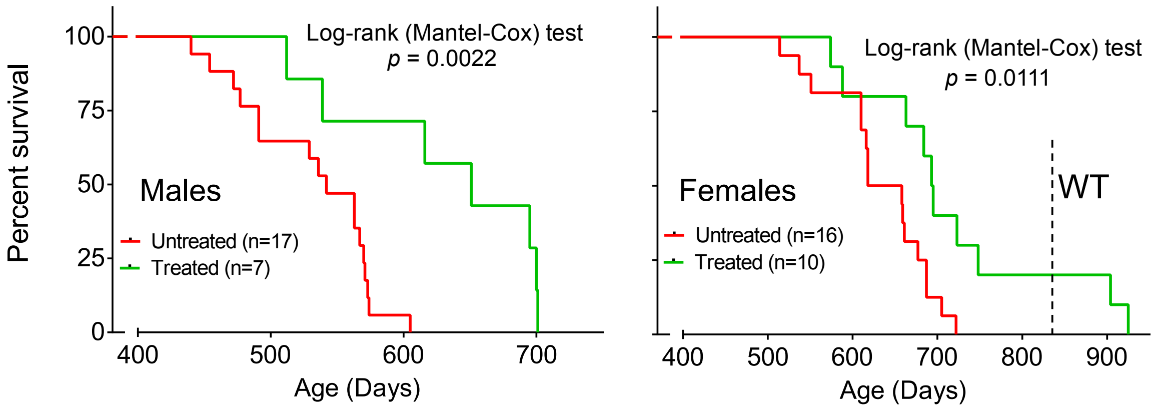

Supplementary Fig. 8 Chronic epichaperome inhibition by PU-AD is non-toxic in mice. **A** Changes in body weight, **B** clinical chemistry parameters and **C** tissue morphology (hematoxylin and eosin stained tissues) of C57/BL6 mice that received PU-AD (n = 7) or vehicle (n = 6) for the duration indicated in panel **A**. Graph, each data point is the mean of an individual mouse group. Scale bars in **C**, 1000µm for lung, brain and heart, 200µm for all other. **D** Kaplan-Meier plots demonstrate that PU-AD significantly extends survival of PS19 mice. WT, average survival for non-Tg mice. Mice were administered PU-AD intraperitoneally at 75mg kg^{-1} on a three-times per week schedule. P value by two-sided log-rank test is shown. Source data are provided as a Source Data file.
