# Supplementary material for: The epichaperome is a mediator of toxic hippocampal stress and leads to protein connectivity-based dysfunction
Source: Nat Commun. 2020 Jan 16;11:319. doi: 10.1038/s41467-019-14082-5 (PMC6965647; doi:10.1038/s41467-019-14082-5)
Supplement: Supplementary file 3 — Reporting Summary [file 41467_2019_14082_MOESM3_ESM.pdf]

nature research

Corresponding author(s): Gabriela Chiosis

Last updated by author(s): Dec 5, 2019

Reporting Summary

Nature Research wishes to improve the reproducibility of the work that we publish. This form provides structure for consistency and transparency in reporting. For further information on Nature Research policies, see Authors & Referees and the Editorial Policy Checklist.

Statistics

For all statistical analyses, confirm that the following items are present in the figure legend, table legend, main text, or Methods section.

n/a	Confirmed	
☐	☒	The exact sample size (*n*) for each experimental group/condition, given as a discrete number and unit of measurement
☐	☒	A statement on whether measurements were taken from distinct samples or whether the same sample was measured repeatedly
☐	☒	The statistical test(s) used AND whether they are one- or two-sided<br>*Only common tests should be described solely by name; describe more complex techniques in the Methods section.*
☐	☒	A description of all covariates tested
☐	☒	A description of any assumptions or corrections, such as tests of normality and adjustment for multiple comparisons
☐	☒	A full description of the statistical parameters including central tendency (e.g. means) or other basic estimates (e.g. regression coefficient) AND variation (e.g. standard deviation) or associated estimates of uncertainty (e.g. confidence intervals)
☐	☒	For null hypothesis testing, the test statistic (e.g. *F*, *t*, *r*) with confidence intervals, effect sizes, degrees of freedom and *P* value noted<br>*Give P values as exact values whenever suitable.*
☒	☐	For Bayesian analysis, information on the choice of priors and Markov chain Monte Carlo settings
☒	☐	For hierarchical and complex designs, identification of the appropriate level for tests and full reporting of outcomes
☐	☒	Estimates of effect sizes (e.g. Cohen's *d*, Pearson's *r*), indicating how they were calculated

Our web collection on statistics for biologists contains articles on many of the points above.

Software and code

Policy information about availability of computer code

Data collection	LC-MS data: Maxquant version 1.6.0.13; Fluorescence measurements: Molecular Devices SpectraMax i3 fluorescence plate reader; spatial memory and locomotion using Barnes maze (San Diego Instruments; model #7001–0235) and Openfield (Actitrack, Panlab, Barcelona, Spain), respectively. All the videos were recorded and analysed for distance (m) and speed (m/s) using the ANY-maze Video Tracking System (v4.94h beta, 4.112. Stoelting Co). electrophysiology: Data were monitored and acquired using Axograph X software. Data analysis was performed using Axograph X built-in analysis and IGOR Pro software (Wavemetrics); Digital autoradiography was performed by placing tissue sections in a film cassette against a phosphor imaging plate (Fujifilm BAS-MS2325; Fuji Photo Film) for a 4-day exposure period at −20°C. Phosphor imaging plates were read at a pixel resolution of 25 µm with a Typhoon 7000 IP plate reader (GE Healthcare). After autoradiographic exposure, the same frozen sections were then dried and darkfield images were taken on an Observer Z1 microscope (Carl Zeiss, Germany) with 5x/0.15NA objective and ZEN2.3 acquisition software. For epichaperome analysis and quantification, internal standards were included in each autoradiography cassette and image analysis was performed using ImageJ 1.48v. Fluorescence polarization and chemiluminescence: Measurements were performed on a Molecular Devices SpectraMax Paradigm instrument (Molecular Devices, Sunnyvale, CA), and data were imported into SoftMaxPro6 and analysed in GraphPad Prism 7. PET-CT was performed using an integrated PET-CT scanner (Discovery DSTE, General Electric). western blots: The chemiluminescent signal was visualized with Enhanced Chemiluminescence System (GE Healthcare) following manufacturer's instructions and quantified using image Studio Lite Ver. 5.2 (LI-COR Biosciences). Metamorph Software (Molecular Devices, PA) was used to quantify IHC slides. Regions of study were designated by using Panoramic Viewer Software (3DHistech Ltd, Budapest, Hungary); LC-MS/MS to measure concentrations of PU-AD (6410, Agilent Technologies)
Data analysis	For computational analyses and visualization, the following R packages were used: gplots, ggplot2, ReactomePA, clusterProfiler, org.Hs.eg.db, Venndiagram, splitstackshape, plyr, biomaRt, preprocessCore, stats. For network visualization, Cytoscape (version 3.6) was used. Prism v6 was used for statistical testings (t-tests and ANOVA). ImageJ (versions 1.4 and 1.52) was used for western blot quantification. Details about statistics are reported in the Source Data file.

For manuscripts utilizing custom algorithms or software that are central to the research but not yet described in published literature, software must be made available to editors/reviewers. We strongly encourage code deposition in a community repository (e.g. GitHub). See the Nature Research guidelines for submitting code & software for further information.

Data

Policy information about availability of data

All manuscripts must include a data availability statement. This statement should provide the following information, where applicable:

- Accession codes, unique identifiers, or web links for publicly available datasets
- A list of figures that have associated raw data
- A description of any restrictions on data availability

LC-MS data, in total 647 raw files and peak files, that support the findings of this study have been deposited in MassIVE with the MSV000083484 accession number [ftp://massive.ucsd.edu/MSV000083484/]. The iGSEA code and the Cytoscape files have been deposited in GitHub with the accession code chiosislab/Chaperomics_AD_2019 [https://github.com/chiosislab/Chaperomics_AD_2019]. Datasets associated with chaperomics analyses are available in the Supplementary Information as Supplementary Data 1 through 6. The source data underlying all main and supplementary figures are provided as a Source Data file.

Field-specific reporting

Please select the one below that is the best fit for your research. If you are not sure, read the appropriate sections before making your selection.

☒ Life sciences ☐ Behavioural & social sciences ☐ Ecological, evolutionary & environmental sciences

For a reference copy of the document with all sections, see nature.com/documents/nr-reporting-summary-flat.pdf

Life sciences study design

All studies must disclose on these points even when the disclosure is negative.

Sample size	No statistical methods were used to pre-determine sample sizes but these are similar to those generally employed in the field.
Data exclusions	No samples were excluded from analyses.
Replication	Several alternative methods were used to validate observations. Experiments were also replicated through multiple cohort analyses. Results shown are representative of several independently performed experiments (see figure legends). There were no findings that could not be replicated or reproduced.
Randomization	No preestablished selection criteria were used, other than gender, phenotype and age. For mouse studies, all mice in corresponding cages, at the appropriate age, were included and animals were assigned randomly to cohorts.
Blinding	Experiments described in Figs. 2B, 3E, 7B-D, 8B-D and Supplementary Figs. 4-8 were randomized and investigators were blinded to allocation and outcome assessments. All other experiments were not randomized and investigators were not blinded.

Reporting for specific materials, systems and methods

We require information from authors about some types of materials, experimental systems and methods used in many studies. Here, indicate whether each material, system or method listed is relevant to your study. If you are not sure if a list item applies to your research, read the appropriate section before selecting a response.

Materials & experimental systems

n/a	Involved in the study	
☐	☒	Antibodies
☐	☒	Eukaryotic cell lines
☒	☐	Palaeontology
☐	☒	Animals and other organisms
☐	☒	Human research participants
☐	☒	Clinical data

Methods

n/a	Involved in the study	
☒	☐	ChIP-seq
☒	☐	Flow cytometry
☒	☐	MRI-based neuroimaging

Antibodies

Antibodies used	All antibodies and relevant information is provided in the Methods .
Validation	All antibodies are commercially available and have been validated by the manufacturer. Supporting publications are found on the manufacturer's site. Relevant positive and negative controls were used to further validate several antibodies as indicated in the relevant figures.

Eukaryotic cell lines

Policy information about cell lines

Cell line source(s)	N2a cells were purchased from ATCC. Existing and well-described human iPSC lines were used in this study, representing healthy controls (CV4a, NDC5; described in 62,63), familial AD caused by a duplication of the APP gene on chromosome 21 (APPdp; described in 62) or APP KM670/671NL (Swedish mutation; APPswe 2.1, 1.2 lines; described in 64), or sporadic AD (SAD2, SAD7; described in 63).
Authentication	Cell were authenticated using short tandem repeat profiling
Mycoplasma contamination	Cells were routinely tested for mycoplasma and were found to be negative
Commonly misidentified lines (See ICLAC register)	none was used

Animals and other organisms

Policy information about studies involving animals; ARRIVE guidelines recommended for reporting animal research

Laboratory animals	Two separate cohorts of transgenic mice were used in this study: (1) male and female hemizygous PS19 harbouring P301S mutant human tau31 and (2) male and female 3xTg.58 The PS19 strain breeds as hemizygotes and the non-transgene containing offspring were used as control animals for transgenic littermates. The 3xTg model was generated using mice of a mixed background; C57BL/6 and 129S1/Sv. Some studies have used as control mice the original background strain provided by Dr. Frank LaFerla (C7BL/6;129X1/SvJ;129S1/Sv). In our study, a separately bred strain of wild type mice, B6129SF2/J, of an identical mixed background were used to control for 3xTg experiments.59 PS19 mice were bread and housed with same-sex littermates at Memorial Sloan Kettering Cancer Center (MSKCC). 3xTg mice were purchased from The Jackson Laboratories and housed at MSKCC. Pharmacokinetic studies were performed in male B6D2F1 mice (Harlan Laboratories). Male and female C57/BL6 mice were used for the toxicology study. Mice were used at the ages indicated throughout the manuscript.
Wild animals	The study did not involve wild animals
Field-collected samples	the study did not include samples collected from the field
Ethics oversight	All procedures were approved by the MSKCC Institutional Animal Care and Use Committee.

Note that full information on the approval of the study protocol must also be provided in the manuscript.

Human research participants

Policy information about studies involving human research participants

Population characteristics	Patients have established diagnosis of mild-moderate Alzheimer's Disease by board-certified neurologist (MSKCC or non-MSKCC) based upon neurological and neuropsychological evaluation following the National Institute on Aging- Alzheimer's Disease Association criteria that recently revisited the NINCDS-ADRDA criteria
Recruitment	Ages Eligible for Study: 21 Years to 90 Years (Adult, Older Adult)<br>Sexes Eligible for Study: All<br>Documentation of diagnosis of mild-moderate Alzheimer's disease, as above, by board-certified neurologist.
Ethics oversight	The microdose 124I-PU-AD PET-CT (Dunphy, M. PET Imaging of Subjects Using 124I-PU-AD available from: http://clinicaltrials.gov; NCT03371420) was approved by the institutional review board, and conducted under an exploratory investigational new drug (IND) application approved by the US Food and Drug Administration.

Note that full information on the approval of the study protocol must also be provided in the manuscript.

Clinical data

Policy information about clinical studies

All manuscripts should comply with the ICMJE guidelines for publication of clinical research and a completed CONSORT checklist must be included with all submissions.

Clinical trial registration	ClinicalTrials.gov Identifier: NCT03371420
Study protocol	https://clinicaltrials.gov/
Data collection	124I-PU-AD tracer was synthesized in-house by the institutional cyclotron core facility at high specific activity. Analyses of the epichaperome by positron emission tomography were performed as previously reported.24 In brief, research PET-CT was performed using an integrated PET-CT scanner (Discovery DSTE, General Electric). CT scans for attenuation correction and anatomic coregistration were performed before tracer injection. Patients received 185 megabecquerel (MBq) of 124I-PU-AD by peripheral vein over two minutes. PET data were reconstructed using a standard ordered subset expected maximization iterative

algorithm. Emission data were corrected for scatter, attenuation, and decay. 124I-PU-AD scans (PU-AD PET) were performed at 3 h after tracer administration.

Outcomes

Positive PET image in disease relevant brain regions
