# Supplementary material for: The epichaperome is a mediator of toxic hippocampal stress and leads to protein connectivity-based dysfunction
Source: Nat Commun. 2020 Jan 16;11:319. doi: 10.1038/s41467-019-14082-5 (PMC6965647; doi:10.1038/s41467-019-14082-5)
Supplement: Supplementary file 4 — Description of Additional Supplementary Files [file 41467_2019_14082_MOESM4_ESM.pdf]

Description of Additional Supplementary Files

File Name: Supplementary Data 1
Description: Interactome Datasets. Interactome Datasets identified for human AD vs ND (sheet 1), iPSC-derived neurons with a duplication in the APP gene vs control (sheet 2), PS19 mice vs WT mice (sheet 3), N2a+tau vs N2a-vector (sheet 4) and a list of the human chaperome created based on a published dataset (see Methods) (sheet 5).

File Name: Supplementary Data 2
Description: Interactome gene-set enrichment analysis (iGESA). Column description (sheet 1). Functional annotation of the chaperome's interactome is provided in sheet 2, with those functions enriched in AD and ND, in sheets 3 and 4, respectively.

File Name: Supplementary Data 3
Description: Mouse to human orthologues conversion table.

File Name: Supplementary Data 4
Description: SynGO list. GO terms important for synapse biology (as curated by Synapse Gene Ontology and Annotation Initiative, SynGO).

File Name: Supplementary Data 5
Description: Full list of differentially expressed proteins as input for GSEAs analyses.

File Name: Supplementary Data 6
Description: GSEAs analyses output files.
